# The Increasing Importance of Gene-Based Analyses

**DOI:** 10.1371/journal.pgen.1005852

**Published:** 2016-04-07

**Authors:** Elizabeth T. Cirulli

**Affiliations:** Center for Applied Genomics and Precision Medicine, Duke University School of Medicine, Durham, North Carolina, United States of America; Georgia Institute of Technology, UNITED STATES

## Abstract

In recent years, genome and exome sequencing studies have implicated a plethora of new disease genes with rare causal variants. Here, I review 150 exome sequencing studies that claim to have discovered that a disease can be caused by different rare variants in the same gene, and I determine whether their methods followed the current best-practice guidelines in the interpretation of their data. Specifically, I assess whether studies appropriately assess controls for rare variants throughout the entire gene or implicated region as opposed to only investigating the specific rare variants identified in the cases, and I assess whether studies present sufficient co-segregation data for statistically significant linkage. I find that the proportion of studies performing gene-based analyses has increased with time, but that even in 2015 fewer than 40% of the reviewed studies used this method, and only 10% presented statistically significant co-segregation data. Furthermore, I find that the genes reported in these papers are explaining a decreasing proportion of cases as the field moves past most of the low-hanging fruit, with 50% of the genes from studies in 2014 and 2015 having variants in fewer than 5% of cases. As more studies focus on genes explaining relatively few cases, the importance of performing appropriate gene-based analyses is increasing. It is becoming increasingly important for journal editors and reviewers to require stringent gene-based evidence to avoid an avalanche of misleading disease gene discovery papers.

## Disease Gene Discovery Using Next Generation Sequencing

The introduction of next generation sequencing has provided a major step forward in our ability to identify genetic variants influencing human traits. Whereas previous technologies restricted researchers to only certain candidate genes, diseases with clear inheritance patterns in very large families, and common genetic variation, we are now able to routinely assess humans for essentially all of the genetic variation present throughout the genome. This is true for common, low-frequency, and rare variants and even includes de novo mutations, a class that was previously difficult to systematically assess. While most next generation studies have thus far focused on coding variation via exome sequencing, this is evolving as costs decrease and our ability to interpret the non-protein coding portions of the genome improves.

With the wealth of information available from complete sequence data, it can be difficult to wade through all of the potentially interesting genetic variants to home in on the ones actually impacting the trait under study. For example, each person carries millions of genetic variations, hundreds of which are rare and predicted to be damaging. Additionally, numerous genes can be plausibly linked to any particular phenotype. The ease with which many rare coding variants can be logically linked to a phenotype—the “narrative potential” [1,2] of each variant—makes it especially important to interpret sequence data in a rigorous fashion and not overemphasize gene function when strong statistical support is lacking. Unlike the gold standard methods used in genome-wide association studies (GWAS) and linkage analyses [3,4], there is not one universal method for analyzing all next generation sequence data or rare variants, which can make it difficult for various research groups to standardize their techniques and agree upon the level of support required for causality.

**Fig 1:**
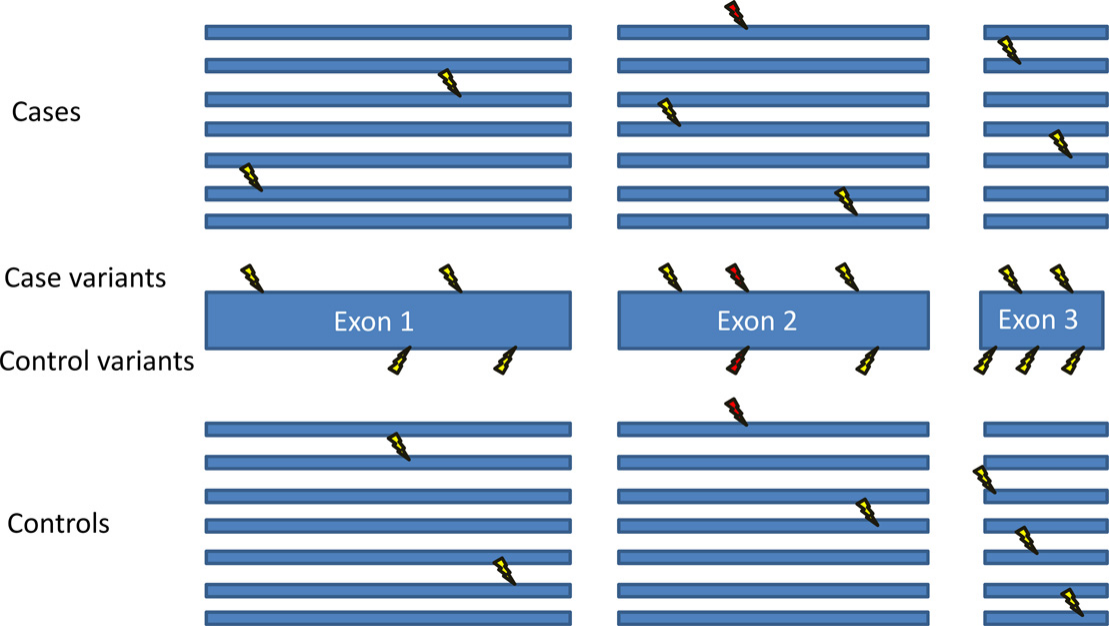
Gene-based collapsing analysis. Shown is the basic schematic for a gene-based collapsing analysis. A gene is completely sequenced in cases and controls, and the number of cases and controls with rare mutations (lightning bolts) in the gene is compared. In contrast, many published articles only use controls to look for the specific variants found in the cases. For example, here the red lightning bolt indicates a variant that is found in both a case and a control. If only case variants are considered, then there are seven cases with different mutations in this gene, and only one of these mutations is found in controls. However, if all variants in the gene are considered, then there are an equal number of cases and controls with mutations in this gene. This collapsing technique can also be used to assess and implicate a gene region instead of an entire gene, for example in S1 Fig and in [16,17].

Nonetheless, a set of guidelines has been put forward that addresses the types of issues to consider in rare variant analysis (Box 1) [1]. These guidelines stress the importance of performing robust statistical analyses when identifying new causal genes. The main process supported by the authors of these guidelines is for all rare variants meeting some criteria in a gene (or genes, or region, or set of regions) to be analyzed together, as each individual variant is too rare to drive a statistical signal on its own. For de novo mutations, a comparison of the case mutation rate against the expected mutation rate for the gene is performed [5–7]. For inherited variants, at the most basic level, a gene-based collapsing or burden analysis can be performed. In this method, a set of criteria define a “qualifying variant” (for example, heterozygous coding variants with minor allele frequency [MAF] < 0.01%). For each gene, each case and each control is then indicated as having or not having at least one qualifying mutation. Finally, a statistical test is performed that compares the proportion of cases with qualifying mutations to the proportion of controls with qualifying mutations (Fig 1). This conceptually simplistic method has successfully identified disease genes in both complex and Mendelian diseases [8–10]. Additionally, numerous more sophisticated gene-based methods have been developed and successfully employed in disease gene discovery that weight variants by function and frequency, including allowing for protective and risk variants to exist in the same gene [11–15].

**Box 1. Key Concepts in this Paper**

Guidelines for interpreting sequence variants: The guidelines referred to in this review come from [1], a recent and highly cited paper authored by many leaders in the field. This paper stresses the importance of statistical analyses when interpreting sequence data.

Gene-based comparison: This method entails completely assessing a gene in all cases and controls used in a study and comparing the variants found in the cases to those found in the controls (Fig 1). For example, each case and each control can be indicated as having or not having at least one “qualifying” mutation in the gene (see below). It is vital that variants found in cases and controls be considered as qualifying based on identical criteria. This method can also be used to group variants in a particular region of a gene or across multiple genes of interest (S1 Fig and [16,17]).

Gene-based statistical analysis: After performing a gene-based comparison, ideally a statistical test is performed to assess the significance of any association between the phenotype and the collection of rare variants found in that gene. Correction for multiple tests is suggested at *p* < 1.7 x 10^−6^ if all genes in the genome are assessed [1].

Qualifying mutations/variants: When analyzing a gene, the researcher must set criteria to determine what variants should be included. The precise criteria will vary depending on the disease and the genetic model, but an example would be all coding variants with MAF < 0.01%. The most important aspect is that case and control variants be assessed in an identical manner.

Significant linkage: When a study analyzes co-segregation between a genomic region and a disease in at least one family with multiple affected individuals and reports a logarithm of the odds (LOD) score above 3.3 [3]. This indicates that a particular region of the genome co-segregates with a trait more often than expected by chance. While I always used a cutoff of 3.3 in this review, the appropriate cutoff can vary based on study design, as detailed in [3].

Data consistent with significant linkage: When a LOD score above 3.3 is not necessarily reported in a study, but the final co-segregation data from all analyzed families presented in the paper appear to be consistent with a LOD score above this threshold. Note that the estimate given in this review will not be correct for all studies, as the final LOD score depends on the specific parameters and criteria used, which are unique to each study.

Functional follow-up: This refers to wet lab experiments that provide additional support for the role of an implicated gene in a disease. The current guidelines recommend the use of stringent statistical procedures in the interpretation of functional follow-up studies to ensure that the observed model phenotypes are not simply due to chance [1]. Here, functional follow-up is defined as per the gene-based experimental evidence entries shown in Table 1 in [1].

ExAC database [18]: This database contains comprehensive summaries of the exome sequence data from more than 30,000 individuals. It is an incredibly useful resource for determining the frequencies of particular variants and can also be used to provide information about how often qualifying variants occur in a particular gene in the general population. This is especially useful for researchers who do not have direct access to large control exome datasets, although the most accurate analyses require cases and controls to be sequenced and analyzed by identical methods.

In contrast to the guidelines’ requirement for gene-based analyses when grouping the effects of rare variants, many published studies do not completely assess implicated genes in controls. These studies instead focus only on the specific rare variants that are found in cases. Such studies identify multiple cases with different mutations in the same gene and then indicate that they have identified a new disease gene because those particular variants are not found in controls. As shown in Fig 1, an appropriate analysis requires an assessment of the entire gene in both cases and controls, determining how often the members of each group have any qualifying mutation in that gene. As the final step, a statistical test is performed to determine whether cases show an enrichment of qualifying variants [1]. It is critical to such an analysis that the variants found in controls be identified and analyzed in the same manner as the case variants. Nonetheless, there are now numerous published studies that identify rare mutations in as few as 2% of the cases and claim to have identified a new disease gene without having determined whether significantly fewer than 2% of controls have similar mutations throughout the gene (for example, [19,20]).

This is not to say that studies that do not screen the entire gene in controls are invalid, but rather that it is more difficult to determine their relevance without this critical comparison to controls. Some signals are sufficiently obvious that they are difficult to misinterpret; for example, multiple patients with very rare recessive variants in a gene, or a gene in which the majority of cases have rare variants. Many of the earliest and highest-profile studies using next generation sequencing to identify causal mutations fall into this latter category, with more than 50% of patients having extremely rare mutations in the same gene (for example, [21–25]). However, much of the low-hanging fruit of this type has already been found, and the requirements for claiming disease gene discovery must be increasingly stringent as the proportion of patients affected by a particular type of mutation decreases.

There are also established guidelines for identifying new disease genes using co-segregation data [3]. Depending on the study design, a logarithm of the odds (LOD) score of 3.3 is generally considered statistically significant evidence of linkage between a region and a disease. However, co-segregation of a specific variant with disease in a single family cannot provide sufficient supporting evidence on its own, as it is difficult to distinguish between multiple potentially disease-causing variants on the same haplotype, some of which may not be observed in the study [1]. Additionally, linkage evidence that is supported by a relatively small number of families or samples can be prone to misleadingly high LOD scores [3]. Nonetheless, the identification of multiple families with different co-segregating variants in the same significantly linked gene can provide strong support for causality [3].

Additional evidence to support the role of a gene in disease can be provided by functional follow-up, including studies in model organisms and cell lines. While such assays can be of extreme importance in determining the mechanisms underlying disease processes, the current guidelines recommend the use of stringent statistical procedures in their interpretation to ensure that the observed model phenotypes are not simply due to chance [1]. An over-reliance on functional data to prove causality can be problematic, as there are a number of pitfalls that require expertise and careful planning to avoid, such as the misinterpretation of model organism phenotypes, the off-target effects of antisense techniques, the potential lack of appropriate controls, and the use of inappropriate genetic models, such as a homozygous knockout in a mouse when the implicated human variant is a heterozygous amino acid change [1,2,26–29].

## An Assessment of Trends in the Literature

Here, I review 150 papers that use exome sequence data to discover that different rare variants in a particular gene can cause the same disease or phenotype (S1 Table). I restrict to papers that focus on a dominant model of inheritance (including de novo mutations) and study germline as opposed to somatic mutations. Because of my focus on combining the effects of rare variants, I require the paper to present at least two unrelated cases with qualifying mutations at different sites in the same gene. For each paper, I have evaluated whether it performs a gene-based comparison by assessing the frequency of control variants throughout the gene (or gene mutation rate for studies of de novo variation), whether it uses a gene-based statistical analysis to support its results, whether it reports a LOD score above the significance threshold of 3.3, and whether the presented data from all analyzed families seem consistent with a final LOD score above 3.3, regardless of whether a final LOD score is actually reported [1,3].

**Fig 2:**
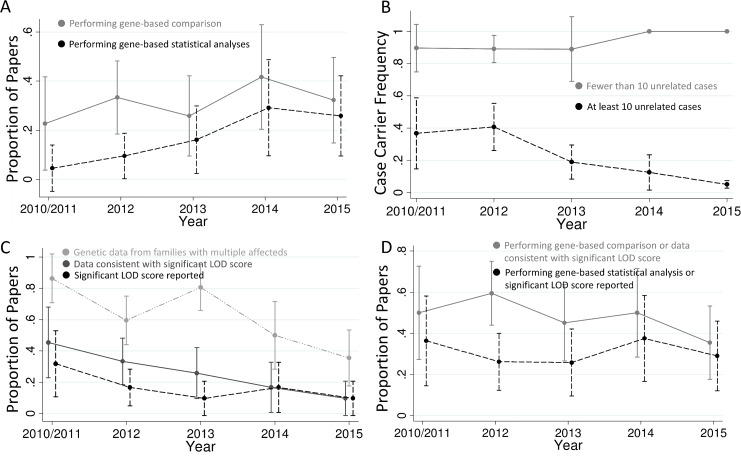
Trends in exome sequencing disease gene discovery papers. (A) Proportion of studies performing gene-based comparisons and statistical analyses. There has been a trend for more papers to use gene-based comparisons versus controls with time (*p* = 0.44, or 0.18 if restricting to studies with at least ten unrelated cases) and a statistically significant increase in the proportion of papers using gene-based statistical analyses (*p* = 0.008, or 0.001 if restricting to studies with at least ten unrelated cases). (B) Proportion of cases with qualifying variants in each implicated gene. Studies with fewer than ten unrelated cases have continued to have a very high proportion of cases with qualifying variants in the implicated gene, while studies with more cases have had a progressively lower proportion of cases with qualifying variants over time (*p* = 0.009 for change over time). (C) Proportion of studies that include co-segregation data from families with multiple affected individuals, that present co-segregation data that appears consistent with a significant LOD score (>3.3), and that report a significant LOD score. The proportion of studies in all three of these categories has been decreasing with time (*p* = 0.001, 0.002, and 0.08, respectively), and the proportion of studies with co-segregation data that present data consistent with a significant LOD score has tended to decrease as well (*p* = 0.06; dropped from 53% in 2010/2011 to 27% in 2015; S5 Fig). (D) Proportion of studies that follow best-practice guidelines [1] in terms of either gene-based analysis or linkage analysis. The less stringent category includes studies that either perform gene-based comparisons or present co-segregation data consistent with significant linkage, and the more stringent category includes papers that perform a gene-based statistical analysis or report a significant LOD score. The total proportion of gene discovery papers following best-practice guidelines does not appear to be changing with time. Plotted are the means and 95% confidence intervals. 2010 and 2011 are merged due to only four studies being from 2010.

Fig 2 shows the trends in the use of these methods over time. While most papers published in 2015 still do not perform statistical analyses of gene-based comparisons, the use of this technique has increased with time (*p* = 0.008; Fig 2A). For example, 40% of the papers published in 2015 used gene-based statistical analyses as compared to 0% in 2010/2011. This effect was largely driven by studies with more than ten unrelated cases (*p* = 0.001). Because diseases with low locus heterogeneity can be easily picked out with a very small sample size, papers with fewer than ten unrelated cases (*n* = 45) tended to have different properties than those with larger sample sizes. Specifically, the proportion of papers with fewer than ten cases that perform gene-based statistical analyses has not increased since 2010. In fact, none of the 21 papers with fewer than ten cases published in 2013–2015 used gene-based statistical analyses (S2 Fig). These studies have also maintained a very high proportion of cases with qualifying variants in the implicated gene, with a mean of 86% in 2010/2011 and 100% in 2015 (Fig 2B). In contrast, larger studies have implicated genes affecting a smaller and smaller proportion of cases over time (*p* = 0.01), with the mean proportion of cases with qualifying variants at 37% in 2010/2011 and falling to 5% by 2015 (Fig 2B). Additionally, the case sample sizes for large studies have been significantly increasing (*p* < 0.001), from a mean of 106 in 2010/2011 to 769 in 2015 (S3 Fig).

In contrast, studies presenting co-segregation data consistent with a significant LOD score have been decreasing with time (*p* = 0.002), falling from 45% in 2010/2011 to 10% in 2015 (Fig 2C). This decrease is largely due to fewer studies evaluating families with multiple affected individuals (*p* = 0.001), falling from 86% in 2010/2011 to 35% in 2015 (Fig 2C). Surprisingly, 44% of studies showing data consistent with a LOD score of at least 3.3 do not directly report this significant evidence in their paper: 23% do not report a LOD score at all, and the other 21% report LOD scores below this cutoff that include only a portion of the total families.

Overall, the proportion of studies following best practices for disease gene discovery via exome sequencing has not been changing with time (Fig 2D). Only 49% of studies have met the relatively lenient criteria of performing a gene-based comparison or presenting co-segregation data consistent with a LOD score of 3.3, and only 30% have met the more stringent requirements of performing a gene-based statistical analysis or actually reporting a LOD score of at least 3.3. These numbers do not improve much when restricting to studies with ten or more cases (56% meet the more lenient criteria and 34% the stringent) or when restricting to studies that identified qualifying variants in less than 5% of cases (60% for lenient and 37% for stringent). These similar proportions in different types of studies indicate that researchers are not generally using the strength of the signal in their study to determine whether best-practice guidelines need to be followed. The proportion of papers that present data from functional follow-up (as defined in [1]) has also not changed over time (S4 Fig), and studies performing functional follow-up were neither more nor less likely to be ones in which best-practice gene discovery techniques were used.

Finally, I examined the number of variants reported in the ExAC database for each implicated gene, matching my counts to the models used in each paper to select qualifying variants in the cases [18]. For example, a paper reporting on cases with loss of function variants with MAF < 0.5% was matched to the total number of stop gain, frameshift, and splice site variants with MAF < 0.5% in that gene in ExAC. Controlling for coding gene length and the proportion of cases with qualifying variants, there was a trend for more qualifying ExAC variants to be found in the genes from studies that did not use gene-based comparisons (*p* = 0.03). While the signal overall was not strong, it was concentrated in the 32 studies reporting fewer than 5% of cases with qualifying variants that did not focus on de novo mutations. This association suggests that studies that do not perform gene-based comparisons may be more prone to false positive discoveries, and gene-based analyses are most crucial when a gene is implicated in a relatively small proportion of cases. As most genes have rare (MAF < 0.005%–0.1%) coding variants in less than 1% of the population [8,18], it makes sense that reasonably sized studies reporting qualifying variants in more than 5% of cases will generally have identified a significant enrichment even if they do not perform an appropriate comparison with controls. Note that there was no statistically significant difference between the ExAC counts for the papers that did and did not use gene-based statistical analyses; the signal was only seen when comparing papers that did and did not use any gene-based comparison. This result indicates that the most important step for avoiding false discoveries thus far has been simply investigating the proportion of controls with qualifying variants in the gene. However, the proportion of cases with qualifying mutations in disease gene discovery papers has been decreasing (Fig 2B), with more than 40% of the studies published in 2014 and 2015 having fewer than 5% of cases with qualifying variants in the implicated gene. Fewer than 50% of these studies used gene-based comparisons with controls, and fewer than 10% provided co-segregation data consistent with a LOD score above 3.3. The importance of using gene-based analyses is therefore increasing, and going forward, this will be a crucial area for researchers to ensure they are appropriately pursuing in their studies [30].

## Considerations when Interpreting Exome Sequencing Studies

**Fig 3:**
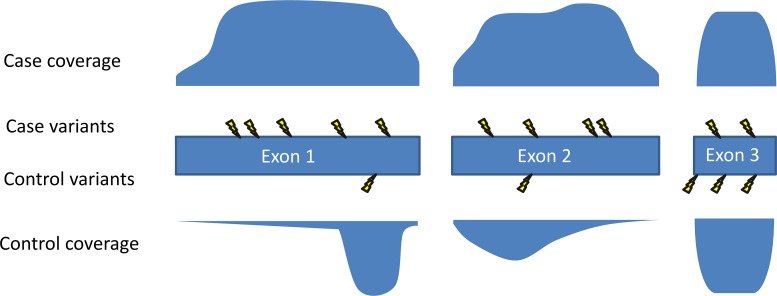
Coverage imbalances can create false signals. The coverage profile of each exon of the gene is shown for cases and controls, with greater filled-in area indicating higher coverage. Mutations are shown with lightning bolts. In this example, the amount of the gene well covered in the cases is much higher than the amount well covered in the controls. As fewer variants are called in regions with poor coverage, the coverage pattern in this gene makes it predisposed to showing more mutations in cases than in controls. One method that can be used to reduce this problem is to prune exons or regions from analysis that show a high case-control imbalance in their coverage patterns [8].

There are many potential sources of error, test statistic inflation, and misinterpretation in gene-based analyses of rare variants. The best practice for such analyses would require a large set of controls who were sequenced and processed using the same methods as the cases [1]. Unfortunately, many research groups do not have access to such a control dataset, but publically available databases like ExAC [18] can provide a reasonable comparison group to get a feel for how often population controls have variants similar to those seen in the cases. Such a check is recommended as the bare minimum when implicating a new disease gene [1]. Even when using controls sequenced via the same methods as the cases, issues of inadequate correction for population stratification, differential coverage of the assessed genes (Fig 3), and sample size imbalances between cases and controls can result in misleadingly low *p*-values. Because of these issues, even an association with a *p*-value that passes correction for multiple tests may occur due to chance. It is therefore advisable that claims of definitive discovery only be made when it can be shown—for example, by a quantile-quantile (QQ) plot—that there is no inflation of the test results (for example, [8,9]).

For studies of de novo mutations involving trio sequencing, a comparison with control trios is not an essential component as it is for other study designs. However, such studies nonetheless require that one take into account the size of the gene and the number of mutations to be expected by chance [5,6]. As has been pointed out previously [1,5], increasing sample sizes means increasing chances of two samples randomly having de novo mutations in the same gene. Appropriate statistical analyses will therefore be of increasing importance as larger studies allow for the identification of genes that are mutated in relatively few cases.

Because of this review’s focus on the technique of grouping rare variants together into one analysis, I do not cover the plethora of studies that find a mutation of interest in just one case or in just one family (S1 Table). There are inherent difficulties in making a strong statistical claim for causality when working with only a single family or case. I also do not touch on the many studies that describe the effects of a single rare variant in a population. The interpretation of such studies is much more straightforward, as standards for single-variant analysis are well established after a decade of single-variant-based GWAS: account for population structure, use the appropriate genetic model, and correct for multiple tests. Nonetheless, such analyses are more difficult for rare variants than for common due to the potential effects of fine-scale population stratification and the propensity of even modest phenotypic outliers to drive very low *p*-values [15].

An additional difficulty that arises in the literature is the building up of evidence about a gene’s involvement in a disease based on a small number of cases from multiple published studies. For example, a study of a rare disease that identifies mutations in a few cases may build the case for causality by compiling information from previous studies identifying mutations in the same gene (for example, [31,32]). Additionally, tools such as the Matchmaker Exchange allow researchers to compare their data and identify overlaps in phenotypes and implicated genes, facilitating disease gene discoveries that might otherwise have been impossible [33,34]. While this cross-referencing can be a powerful tool for building up sample sizes for rare conditions and making connections between related diseases, comparisons across studies require careful analysis. The overall number of cases considered or reported in the literature who do not have mutations in that gene should be taken into consideration, and, again, it is advisable to perform a comparison with the frequency of similar variants in controls.

Another issue with identifying disease-causing variants is penetrance. Most disease-causing mutations are not expected to cause disease in every person who carries them. Observing an implicated variant in a public database or a control, therefore, does not necessarily rule out the variant as causal, nor does observing some unaffected carriers in a family with the disease. However, mutations with reduced penetrance are more difficult to interpret and require studies with larger sample sizes to obtain statistically significant evidence of their involvement. Adding more transparent information about evidence supporting pathogenicity and phenotypic information about variant carriers in public databases would aid in this often complex interpretation [1], as would the creation of a repository allowing researchers to deposit information about controls who harbor potentially pathogenic mutations in suspected causal genes.

All of the studies reviewed here specifically claimed that the identified mutations caused the disease or trait under study. There are many additional studies that do not claim definitive causality but do highlight a specific gene as a new candidate or a likely cause of the disease (for example, [35–38]). It is generally clear that such papers require additional follow-up studies to solidly implicate the gene, but it is worth reiterating this point, especially if appropriate statistical analyses have not been performed. Finally, numerous studies have been published that report negative or inconclusive findings from their next generation sequencing results. These studies can be a useful contribution to the field, especially when they use appropriate statistical analyses (for example, [39–41]).

## Conclusion

The appropriate interpretation of next generation sequence data is one of the main challenges in current human genetics research. The ability to make diagnoses or predictions from a person's genome in the clinic and beyond relies heavily upon the functions of genes being reliably annotated. A literature that is burdened with incorrect gene-phenotype associations will make it extremely difficult to identify useful and accurate information about the potential consequences of discovered variants.

Here, I show that the proportion of disease gene discovery papers using gene-based statistical analyses is increasing. Additionally, there are a multitude of studies that perform rigorous statistical analyses of their exome sequence data and do not claim to have discovered new disease genes, because they have suggestive but not definitive evidence. Nonetheless, more than 50% of the exome sequencing papers claiming to have identified new disease genes published in 2015 are still not using best-practice analysis methods, which are of increasing importance as the proportion of cases with a qualifying variant in each newly implicated gene continues to decrease (Fig 2). Finally, we will need a reassessment of the recommended guidelines as genome sequencing studies become more prevalent to ensure that the roles of noncoding variants are appropriately assessed.

## Supporting Information

S1 Fig Example of region-based analysis. Here, equal numbers of cases and controls contain variants in the same gene. However, all of the case variants are clustered within one region of the gene. In this case, a region-based as opposed to gene-based analysis would be more appropriate to determine the significance of the case enrichment.
(TIF)

S2 Fig Proportion of papers with fewer than ten unrelated cases that use gene-based comparisons and gene-based statistical analyses. Plotted are the means and 95% confidence intervals. 2010 and 2011 are merged due to only four studies being from 2010.
(TIF)

S3 Fig Average number of unrelated cases included in studies with at least ten unrelated cases. Plotted are the means and 95% confidence intervals. 2010 and 2011 are merged due to only four studies being from 2010.
(TIF)

S4 Fig Proportion of papers presenting functional follow-up data. Plotted are the means and 95% confidence intervals. 2010 and 2011 are merged due to only four studies being from 2010.
(TIF)

S5 Fig Proportion of papers with co-segregation data from families with multiple affecteds that present significant LOD scores or data consistent with significant LOD scores. Here, the proportion shown is out of the total number of papers with co-segregation data from families with multiple affecteds; papers without families with multiple affecteds were excluded from the total. Plotted are the means and 95% confidence intervals. 2010 and 2011 are merged due to only four studies being from 2010.
(TIF)

S1 Table Literature search and details about papers used in the study. (XLSX)
