# Supplementary figures and images for: The Increasing Importance of Gene-Based Analyses
Source: PLoS Genet. 2016 Apr 7;12(4):e1005852. doi: 10.1371/journal.pgen.1005852 (PMC4824358; doi:10.1371/journal.pgen.1005852)

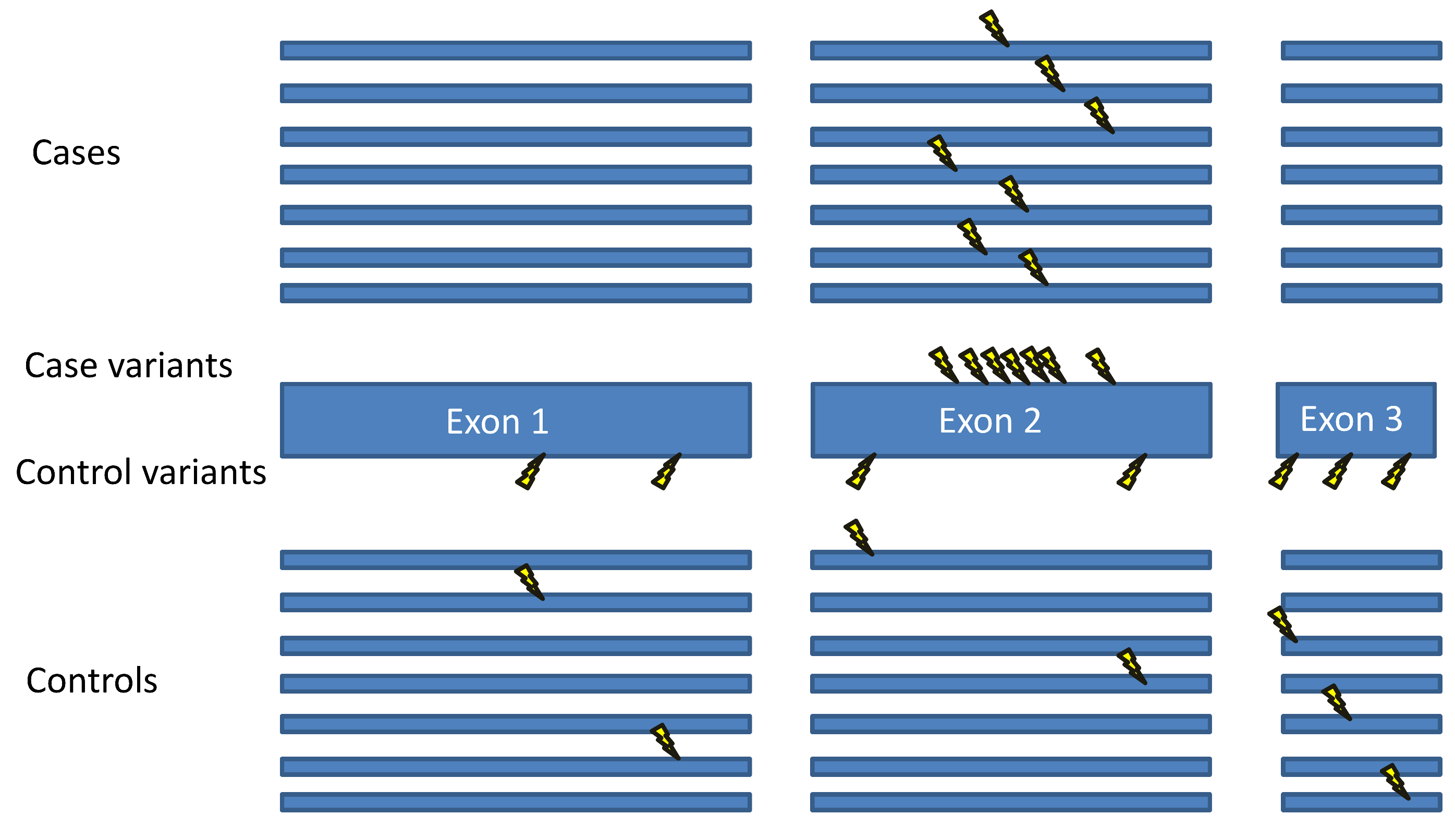

Supplement: S1 Fig — Here, equal numbers of cases and controls contain variants in the same gene. However, all of the case variants are clustered within one region of the gene. In this case, a region-based as opposed to gene-based analysis would be more appropriate to determine the significance of the case enrichment. (TIF) [file pgen.1005852.s001.tif]

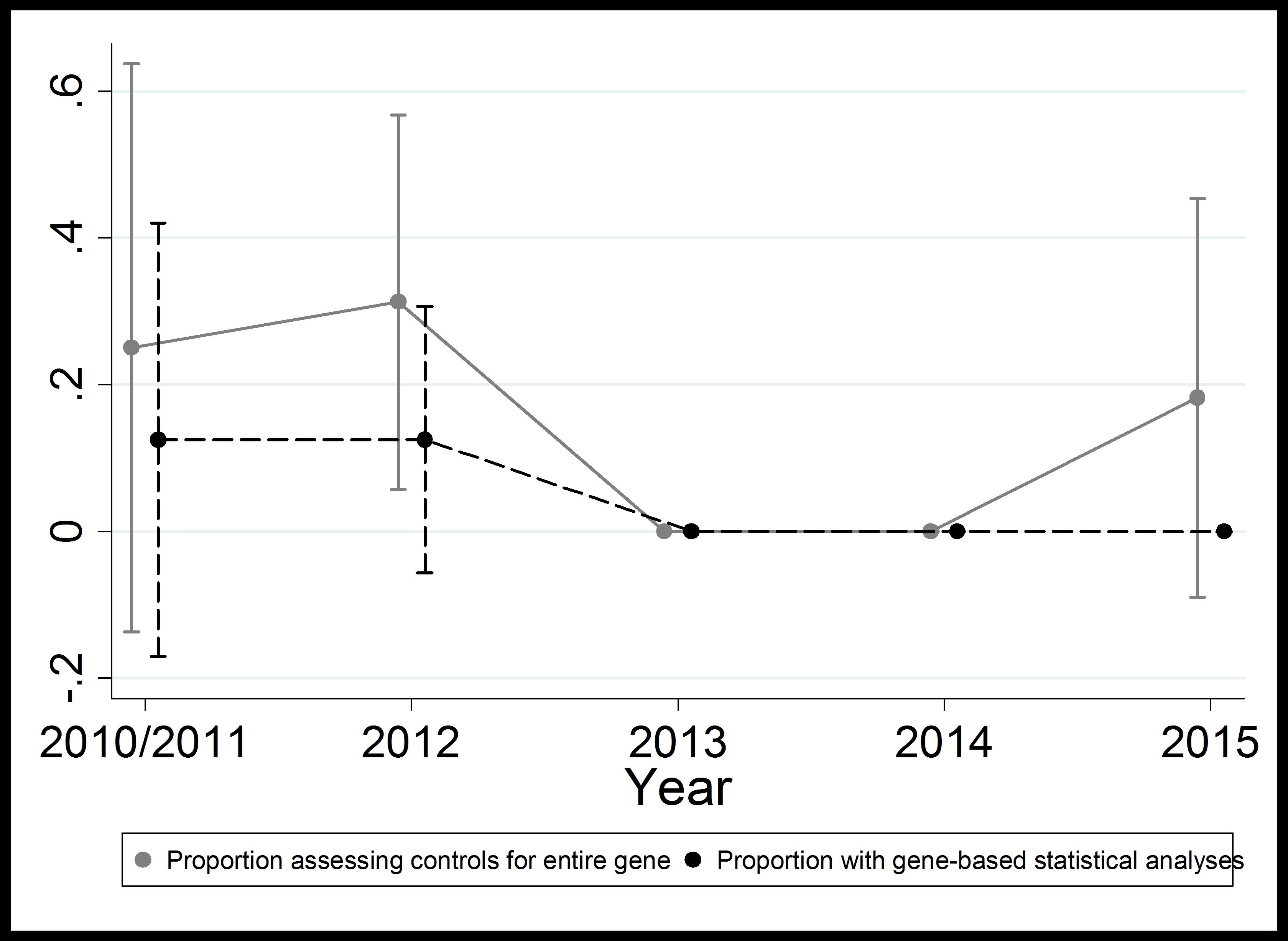

Supplement: S2 Fig — Plotted are the means and 95% confidence intervals. 2010 and 2011 are merged due to only four studies being from 2010. (TIF) [file pgen.1005852.s002.tif]

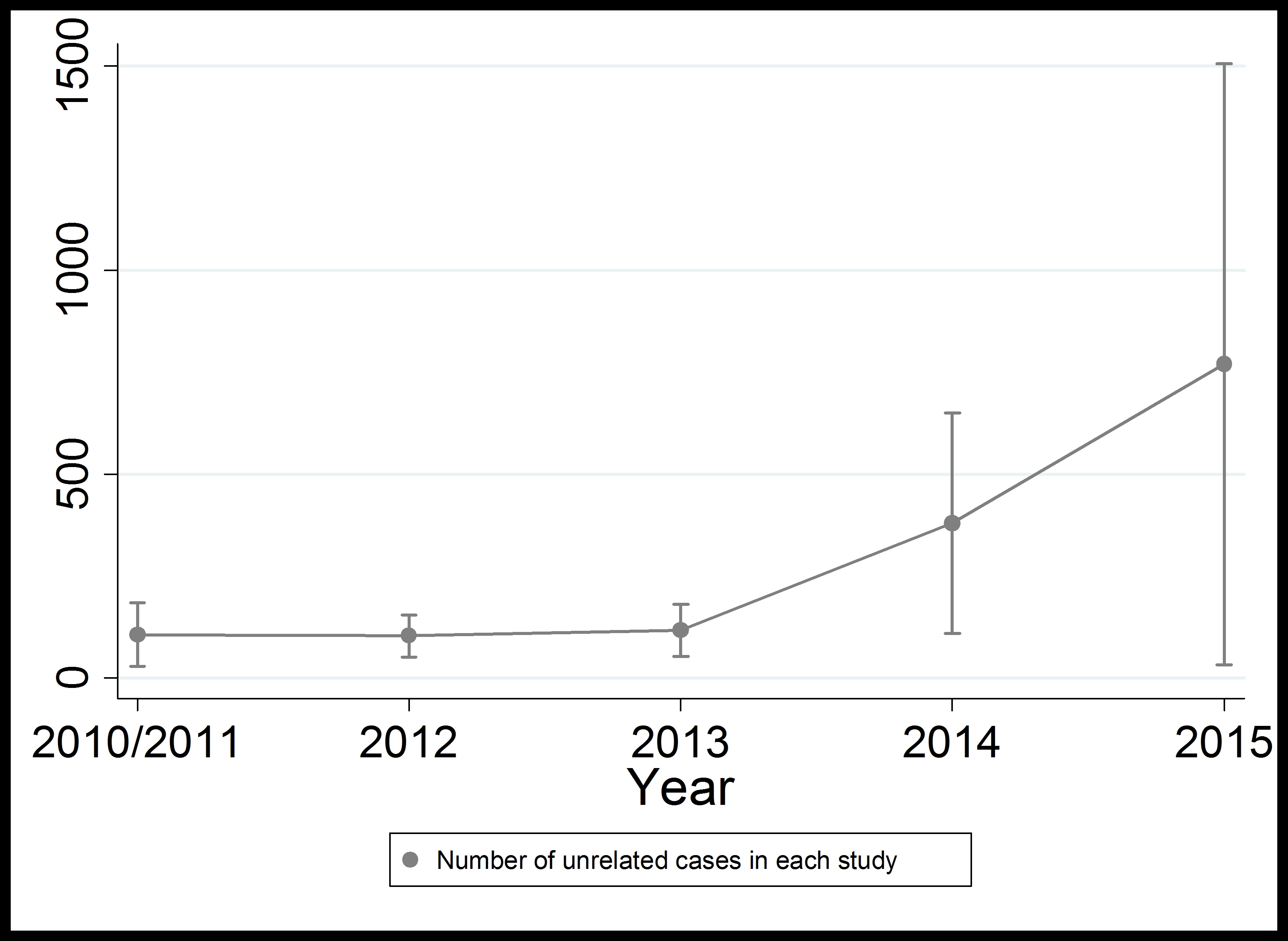

Supplement: S3 Fig — Plotted are the means and 95% confidence intervals. 2010 and 2011 are merged due to only four studies being from 2010. (TIF) [file pgen.1005852.s003.tif]

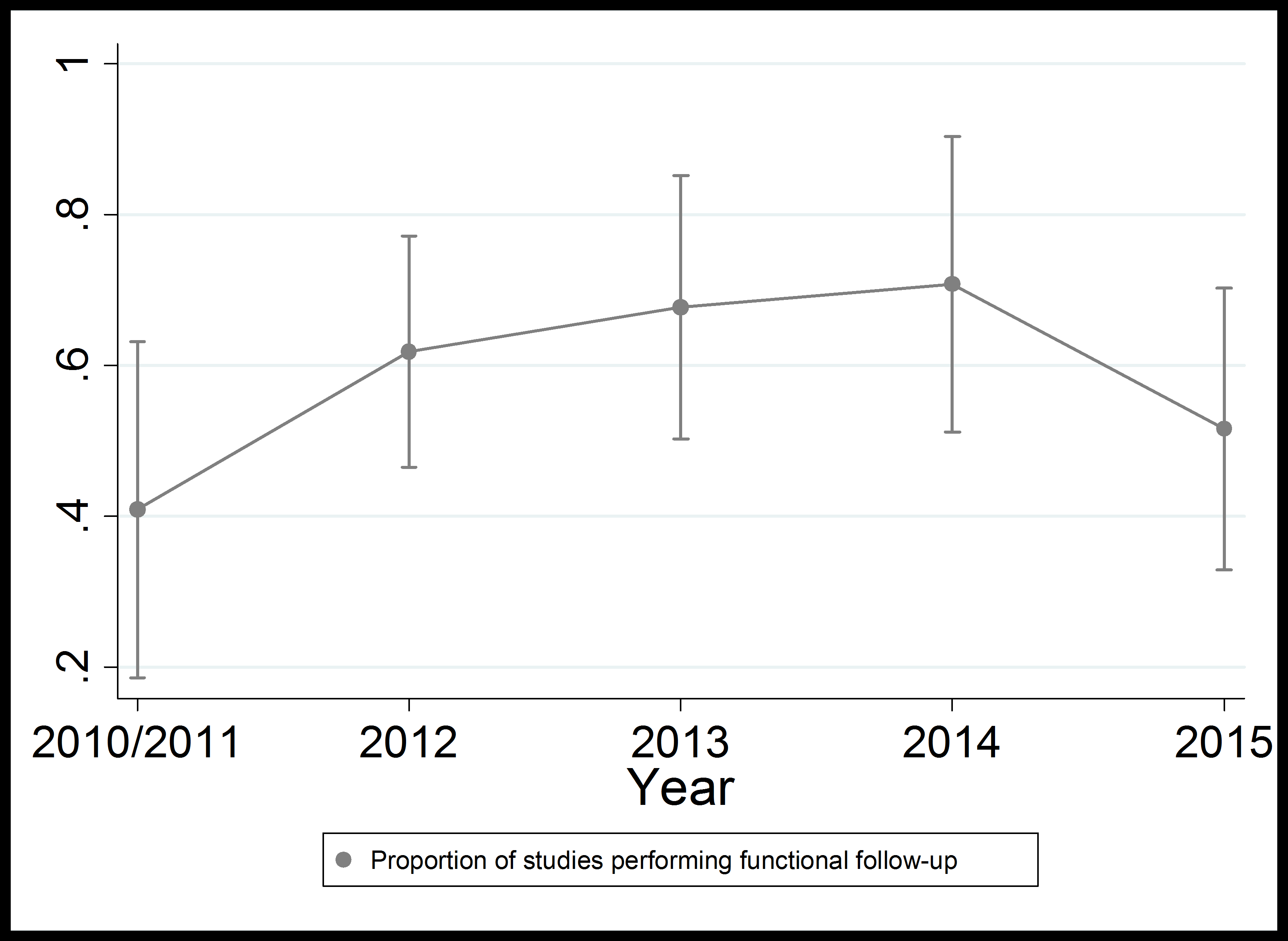

Supplement: S4 Fig — Plotted are the means and 95% confidence intervals. 2010 and 2011 are merged due to only four studies being from 2010. (TIF) [file pgen.1005852.s004.tif]

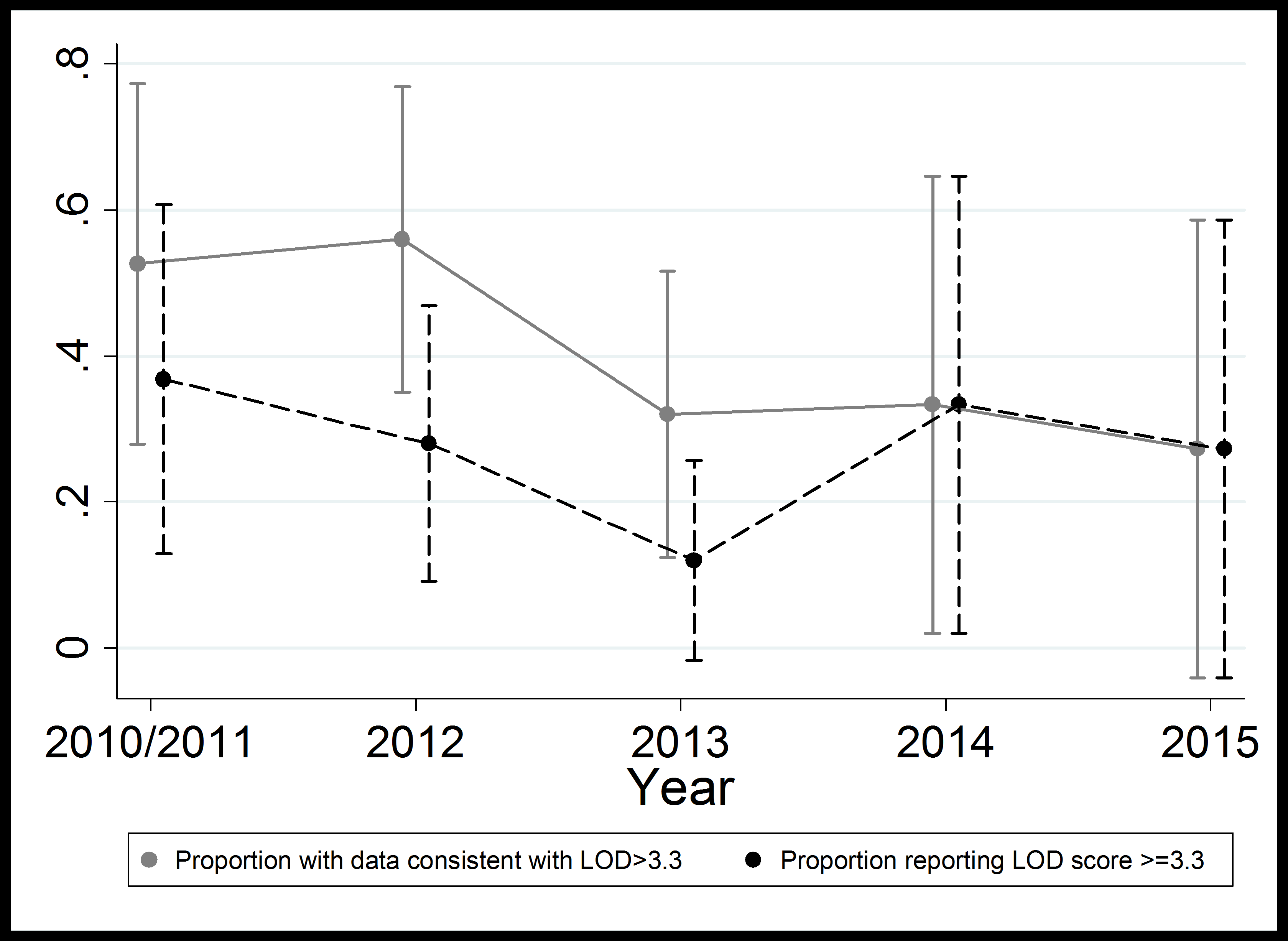

Supplement: S5 Fig — Here, the proportion shown is out of the total number of papers with co-segregation data from families with multiple affecteds; papers without families with multiple affecteds were excluded from the total. Plotted are the means and 95% confidence intervals. 2010 and 2011 are merged due to only four studies being from 2010. (TIF) [file pgen.1005852.s005.tif]
